# A cost-effectiveness analysis of provider interventions to improve health worker practice in providing treatment for uncomplicated malaria in Cameroon: a study protocol for a randomized controlled trial

**DOI:** 10.1186/1745-6215-13-4

**Published:** 2012-01-06

**Authors:** Virginia Wiseman, Lindsay J Mangham, Bonnie Cundill, Olivia A Achonduh, Akindeh Mbuh Nji, Abanda Ngu Njei, Clare Chandler, Wilfred F Mbacham

**Affiliations:** 1 Department of Global Health and Development, London School of Hygiene and Tropical Medicine, London, UK; 2 Department of Global Health and Development, London School of Hygiene and Tropical Medicine, London, UK; 3 Department of Infectious Disease Epidemiology, London School of Hygiene and Tropical Medicine, London, UK; 4 Laboratory for Public Health Research Biotechnologies. The Biotechnology Center, The University of Yaoundé I, Box 8094 Yaoundé, Cameroon; 5 Laboratory for Public Health Research Biotechnologies. The Biotechnology Center, The University of Yaoundé I, Box 8094 Yaoundé, Cameroon; 6 Laboratory for Public Health Research Biotechnologies. The Biotechnology Centre, the University of Yaoundé I, Box 8094 Yaoundé, Cameroon; 7 Department of Global Health and Development, London School of Hygiene and Tropical Medicine, London, UK; 8 Laboratory for Public Health Research Biotechnologies. The Biotechnology Center, The University of Yaounde I, Box 8094 Yaounde, Cameroon

**Keywords:** Cost-effectiveness, malaria, Rapid Diagnostics tests (RDTs)

## Abstract

**Background:**

Governments and donors all over Africa are searching for sustainable, affordable and cost-effective ways to improve the quality of malaria case management. Widespread deficiencies have been reported in the prescribing and counselling practices of health care providers treating febrile patients in both public and private health facilities. Cameroon is no exception with low levels of adherence to national guidelines, the frequent selection of non-recommended antimalarials and the use of incorrect dosages. This study evaluates the effectiveness and cost-effectiveness of introducing two different provider training packages, alongside rapid diagnostic tests (RDTs), designed to equip providers with the knowledge and practical skills needed to effectively diagnose and treat febrile patients. The overall aim is to target antimalarial treatment better and to facilitate optimal use of malaria treatment guidelines.

**Methods/Design:**

A 3-arm stratified, cluster randomized trial will be conducted to assess whether introducing RDTs with provider training (basic or enhanced) is more cost-effective than current practice without RDTs, and whether there is a difference in the cost effectiveness of the provider training interventions. The primary outcome is the proportion of patients attending facilities that report a fever or suspected malaria and receive treatment according to malaria guidelines. This will be measured by surveying patients (or caregivers) as they exit public and mission health facilities. Cost-effectiveness will be presented in terms of the primary outcome and a range of secondary outcomes, including changes in provider knowledge. Costs will be estimated from a societal and provider perspective using standard economic evaluation methodologies.

**Trial Registration:**

ClinicalTrials.gov: NCT00981877

## Background

Governments and donors all over Africa are searching for sustainable, affordable and cost-effective ways to improve the quality of malaria case management. Widespread deficiencies have been reported in the prescribing and counselling practices of providers (by which we mean health workers) responsible for treating febrile patients attending public and private facilities [1-8]. Similar problems have been reported in Cameroon where malaria accounts for 35%-40% of all deaths, 50% of morbidity among children under the age of five, 40%-45% of medical consultations and 30% of hospitalizations [9,10].

Despite widespread availability of malaria testing using microscopy in public and private facilities in Cameroon and recent guidelines from the World Health Organization (WHO) recommending parasitological confirmation of suspected malaria cases in all patients before treatment where testing facilities are available [11], symptomatic diagnosis of malaria remains routine in more than 50% of consultations [12]. There is, however, increasing interest in scaling up the use of rapid diagnostic tests (RDTs) to expand access to parasitological diagnosis and improving malaria case management. The Government of Cameroon is piloting the introduction of RDTs into communities in 50 health districts in the national territory [13]. This policy initiative is based on the premise that making RDTs available will make it quicker and easier to test and in turn, promote the rational use of artemisinin-based combination therapy (ACT).

The appeal of RDTs lies in their high specificity and sensitivity. They are relatively simple to use compared with microscopy and do not require specialised skills or laboratory equipment and reagents that are often unavailable in rural or resource poor settings [14]. RDTs are also seen as the solution to malaria over-diagnosis, a practice that can be costly [15] and result in poorer health through delays in access to treatment of the correct diagnosis and repeated treatment seeking costs [16]. Misdiagnosis may also contribute to increasing antimalarial drug pressure and thus resistance, thereby speeding up the ineffectiveness of available and affordable drugs [17]. Hence there is both a human and an economic case for introducing RDTs compared with existing presumptive treatment. However, in order for the full benefits of RDTs to be realised, supporting interventions that encourage health workers to deliver treatment that is consistent with malaria guidelines are likely to be needed.

The diagnosis and subsequent treatment of malaria is a complex decision making process [18]. Interventions must be sympathetic to a wide range of issues that providers face including a lack of training in the use of RDTs especially among more junior staff [19], a distrust of test results particularly negative ones [6,20,21], lack of alternative drugs with which to treat fever patients [19,22] and patient demand for inappropriate medicines [8,19,23]. All of these issues have been shown to affect whether a malaria test is done and in turn acted upon.

If diagnostic and prescribing practices of providers are to be improved through the large-scale procurement and deployment of RDTs and ACTs in countries such as Cameroon, some level of supporting interventions are likely to be needed, or the intended benefits of these investments may be seriously undermined. The WHO recommends that a number of conditions are in place before integrating and scaling up the use of RDTs in malaria control and primary health care services including provider training, monitoring how the test is used and the establishment of clear guidelines that incorporate a diagnosis and treatment algorithm that includes RDTs [24]. To date, the most cost-effective composition of training for providers is not known. There are arguments for both a basic introduction to the tests that will require few resources to implement, and for a more comprehensive programme that not only equips providers with the knowledge of the malaria guidelines and skills to use RDTs, but also strives to improve the quality of malaria case management by supporting providers to change their practice and manage patient expectations, especially when the malaria test is negative. This study will use a cluster randomized controlled trial to help identify, in routine health facility scenarios, which of these options is most effective and cost-effective in equipping providers with the knowledge and practical skills needed to effectively diagnose and treat febrile patients. It is important to compare these options for supporting RDT introduction with current practice in the absence of RDTs but where microscopy is widely available, in order to identify whether there is a value to introducing these new tests at all.

A strength of this trial is that the chosen provider interventions target several specific problems identified through our own formative research that undermine the implementation of malaria treatment guidelines in Cameroon. Between May and November 2009, a cross-sectional cluster survey and series of focus group discussions were conducted to understand current practices in delivering malaria treatment in the two sites targeted for the evaluation. It was revealed that all mission and almost 90% of public health facilities have microscopy testing available, though only about a third used it. Quinine, which should be reserved only for cases of severe malaria, was often used for the treatment of uncomplicated malaria. Factors affecting providers' choice of treatment appeared to be broader than simple consideration of the test result, with many patients receiving antimalairals they do not need. Some of the issues identified were unique to the local setting while others reflect problems experienced across the country and elsewhere. A description of the methods and results of the formative research have been published elsewhere [12,25].

Finally, this study also makes an important contribution to the pursuit of efficiency. While evaluations of a wide range of provider training interventions have been reported in the literature [26-28] using an equally wide range of methods, few of these enable the assessment of the relative value for money of these interventions. This study will provide much needed information on the cost-effectiveness of the selected provider training interventions which will aid health care planners in their decisions over how to allocate scarce health care resources.

## Methods/design

This study is a 3-arm stratified cluster randomised controlled trial across 47 health facilities in two areas of Cameroon. The intervention is being delivered at the facility level and therefore this will be the unit of randomisation with study site as the stratum. Outcomes will be assessed through exit interviews with patients as well as health facility surveys. Economic and financial costs will also be measured to enable the calculation of incremental cost-effectiveness ratios. Ethical approval for this study was obtained from the Cameroon National Ethics Committee and the London School of Hygiene and Tropical Medicine.

### Study area and participants

The two study sites are Yaoundé and Bamenda in the Centre and Northwest regions respectively. The Bamenda study site consists of an urban health district and seven rural health districts that lie within a 21 km radius. It is predominantly an English and pidgin-English speaking region with an estimated population of 2 million. The Yaoundé study site encompasses seven urban health districts and has an estimated population of 2.5 million that is predominantly French-speaking.

Although both study sites lie within the forest ecological zone of Cameroon favorable for the development of the *Plasmodium *parasite and Anopheles vector, they have different climatic patterns. The Yaoundé study site has two main seasons: the long wet season that lasts from February to November (with more intense rains between September and November) and a short dry season from December to January. Transmission in this site is perennial with an inoculation rate of over 100 infected bites per person per month. The Bamenda study site is characterized by one long rainy season (March - October) of intense transmission with inoculation rates of 20 infected bites per person per month. In 2004, the forest ecological zone accounted for 40.6% of the total malaria morbidity (40.1%) recorded in the general population [29,30].

All public and mission health facilities have been enumerated and GPS mapped. Health facilities were informed of the proposed study and asked to give verbal consent before GPS coordinates were obtained. They include public district hospitals and health centres, mission hospitals and mission health centres. The health centres are staffed by nurses and sometimes medical doctors. Each of these health facilities has a propharmacy with a pharmacy attendant and a laboratary for simple diagnostic procedures including microscopy testing.

Facilities will be selected at random within each stratum from those that are not included in the Government pilot roll-out of RDTs, do not solely offer specialist services, see 4 or more febrile patients per day, and are accessible by road throughout the wet season. Selected facilities will be asked to give written consent prior to randomisation. If facility-level consent is not provided replacement facilities will be randomly selected from the remaining list of eligible facilities.

Contamination may occur if providers that have received basic or enhanced training meet to discuss their training or if they meet with providers from the control facilities. This may result in information or strategies being shared, the effect of the intervention spreading to control clusters and possible dilution of differences between treatment arms. In order to reduce the risk of contamination, the different intervention and control facilities are separated by a buffer area. Specifically, facilities within the same health area will be selected if they are ≥ 2 km from another facility in Bamenda and ≥ 1 km in Yaoundé.

All patients (or their caregiver) attending the health facilities will be approached on exit for consent to participate in an exit survey and screened for their eligibility. Patients will be eligible if they are present at the facility and they (or their caregiver) report seeking treatment for fever or suspected malaria. Patients will be excluded if they are pregnant, less than 6 months old or have signs and symptoms of severe malaria. All providers that are responsible for diagnosis and treatment of suspected cases of malaria will be eligible to participate in the provider survey.

Interviewers will explain to all participants that involvement in the study is voluntary and they have the right to withdraw at any point in time and ask any questions. Information about the study will be read to all participants and provided in hard copy. All participants will be asked if they give their consent to take part in the study and if so, asked to sign the standard consent form.

### Interventions

Health facilities will be randomised to either current practice or one of the two provider interventions. The basic intervention is the introduction of RDTs with basic provider training on malaria diagnosis and treatment while the enhanced intervention will be the introduction of RDTs with enhanced provider training. The enhanced training covers the material in the basic training and also strives to improve the quality of care by supporting providers to adapt their practice by encouraging further discussion of the malaria guidelines, interactive self-awareness, improve their ability to communication with patients and colleagues.

#### Supply of RDTs

Facilities randomised to either the basic intervention or the enhanced intervention will be supplied with RDTs for use in diagnosing malaria. The RDT that will be used is SD Bioline Malaria Ag Pf/Pan which is able to detect *P. falciparum, P.vivax, P. malariae or P.ovale*. This test was chosen in conjunction with the National Malaria Control Programme and is reported to have a minimum detection rate of for *P. falciparum *of 97.5% even at low levels of parasitaemia (200 parasites/μl) [31].

The supply of RDTs is intended to be relatively stable in order to assess the impact of the two provider interventions in the context of a reliable supply system. RDTs will be provided by the study team, free of charge, on a four-weekly rotation basis. Estimates of RDTs required will be determined in discussion with the facility head and based on routine records of the number of febrile patients that a facility can expect during a month (taking into account seasonal variations). Members of the research team will deliver RDTs to the facilities at the start of each month with the option for replenishment between delivery dates. Stock management records will be kept by the study team to monitor the distribution of these RDTs.

Facilities will be requested not to charge for the use of an RDT in children <5 years, but will be able to charge a token fee of at most 100CFA (0.2USD) for all patients above 5 years of age. Currently there is no national policy for the cost of RDTs in health facilities. Facilities are routinely supplied with ACTs and we will not alter the current distribution of medicines by the government or mission authorities. Our formative research found that more than 80% of public and mission facilities had ACTs in stock. In the analysis we will take into account that stock-outs of ACTs would prevent patients from receiving ACTs by also considering a secondary outcome which allows either prescription or receipt of an ACT.

#### Basic Provider Training (BT)

Facilities randomised to the basic intervention will be supplied RDTs and receive basic provider training on malaria diagnosis and treatment. This training is intended to mimic the style of workshop that is routinely implemented as in-service health worker training. The training will be conducted over one day and contain three training modules: 1) Malaria Diagnosis; 2) Rapid Diagnostic Testing; 3) Malaria Treatment. Together these three training modules will provide health workers with the knowledge and skills on why malaria testing is recommended, how to use an RDT, the treatment algorithm and details contained in the malaria guidelines. The malaria guidelines state how confirmed cases of uncomplicated malaria should be treated, including advice on dosing and treatment regimens for different types of ACT. The training also provides advice on other causes of febrile illness which should be investigated if the malaria test is negative. The module on RDTs includes a practical session in which all health workers will get hands-on experience of the steps involved in using an RDT.

The training will be conducted in conference halls of health districts located in both study sites. The following types of providers will be invited to the training: medical doctors, nurses, laboratory technicians and pharmacy attendants. Each facility will be invited to select 3 providers to attend the training. The training will be conducted jointly by medical doctors, representatives of the national malaria control programme and the research team. The trainers will receive extensive briefing by the research team and given a trainer's manual which provides detail of the material for each module and how it should be delivered. The training manual also includes standardized power-point presentations. In addition, the trainers will be trained in presentation and communications skills.

Each basic training workshop will train 25-30 providers. The training primarily takes a didactic seminar style in which the trainer delivers the training material, though there is scope for questions and discussion. A participant's training manual will be given to providers that attend the training course and includes all essential reference material including the malaria treatment guidelines. Participants will also be provided with job aids for RDTs and a treatment algorithm to be placed on their tables while in their health facilities.

All participants of the basic training will be strongly encouraged to train others at their facilities using copies of the training materials including manuals, copies of presentations and table top flip charts. This will not be mandatory or enforced, but as an incentive only those that train their colleagues will be given a certificate of completion.

#### Enhanced Provider Training (ET)

All facilities randomized to the enhanced intervention will be supplied RDTs and receive enhanced provider training. Enhanced provider training covers all the material contained in the basic provider training but also additional material targeting improvements in quality of care. The enhanced provider training will last for a total of three days (one day on basic training modules and an extra two days for the additional material). This training is more resource-intensive than routine in-service training, but intends to tackle some of the ingrained factors affecting health worker prescribing in relation to malaria, as identified in Cameroon and elsewhere.

The enhanced provider training contains three additional training modules: 4) Adapting to Change; 5) Professionalism; 6) Communicating Effectively. A specific focus of these modules is to address challenges posed by RDTs for interactions between the health workers and patients. The modules take an interactive and supportive approach to training with the majority of the material covered using small-group work. There are several exercises in each module based on games and puzzles, testimonials on the use of RDTs, self-developed participatory drama and role-playing. In these additional modules the role of the trainer is to direct and facilitate the learning process rather than provide technical information. The participants will be given training materials to accompany these modules.

The adapting to change module seeks to provide health workers with the opportunity to reflect and discuss the clinical guidelines, and learn from others. This module includes testimonials on the use of RDTs and participants have the opportunity to reflect on and discuss the recommendations in the malaria guidelines. As well as small group discussions, the module has a card game that 4-6 participants can play. Participants take turn in collecting cards and achieve a point when they present three cards that show a patient has received treatment in line with guidelines. This can be achieved by presenting a 'patient with fever' card accompanied by a 'RDT positive' card and an 'ACT' card, or alternatively by presenting a 'patient with fever' card accompanied with an 'RDT negative' card and a 'further investigation' card. The game ends when a participant has treated five patients in line with the guidelines and scored five points.

The professionalism module appeals to the providers to identify and agree what values and behaviours are important when providing care. It also emphasises the importance of working as a team and supporting each other. The module includes an exercise that considers real-life scenarios that may interrupt the process of care and participants are encouraged to develop strategies for managing these situations.

The final module focuses on improving the providers' skills in communicating with patients. It starts by reflecting on what patients think about malaria and malaria treatment. The module also focuses on managing patient expectations and allows providers to develop skills and techniques for explaining to patients why they should be tested, and also for the situation when the test is negative and an antimalarial should not be prescribed. Dramas are developed and acted out by the participants with the support of the facilitators to help providers understand the consequences for patients when they are not prescribed the recommended medicine and what alternative courses of action may be pursued.

As with the basic training, all participants of the enhanced training will be strongly encouraged to train others at their facilities and will only be given a certificate of completion once this has been undertaken.

#### Control Arm

The control arm represents current practice. Providers in these facilities will not receive RDTs or training as part of the study and are expected to continue to provide usual medical care for fever patients attending their facility. Our formative research showed that 90% of public health facilities and all mission health facilities in the study sites had microscopy testing, though none had RDTs.

### Objectives

The primary objective is to evaluate the effectiveness and cost-effectiveness of:

• Basic intervention (i.e. introducing RDTs with basic provider training) compared to current practice;

• Enhanced Intervention (i.e. introducing RDTs with enhanced provider training) compared to current practice; and

• Enhanced Intervention compared to Basic Intervention.

Secondary objectives include:

• To describe the process of implementing the interventions including participant assessment of the training received;

• To document health worker knowledge and ability to test and appropriately treat patients with suspected malaria;

• To evaluate patient satisfaction with the quality of care received at the health facility;

• To calculate the economic and financial costs of the provider interventions;

• To assess whether the effectiveness and cost-effectiveness of the interventions varies according to urban/rural residence or socioeconomic status of the patient.

### Hypotheses

• Basic Intervention is more effective in improving the treatment and diagnosis of malaria (measured by adherence to malaria treatment guidelines) than current practice.

• Enhanced Intervention will be more effective in improving the treatment and diagnosis of malaria compared to current practice and compared to Basic Intervention.

• Basic Intervention is more cost-effective in improving the treatment and diagnosis of malaria compared to current practice.

• Enhanced Intervention is more effective and more costly compared to Basic Intervention.

**Figure 1:**
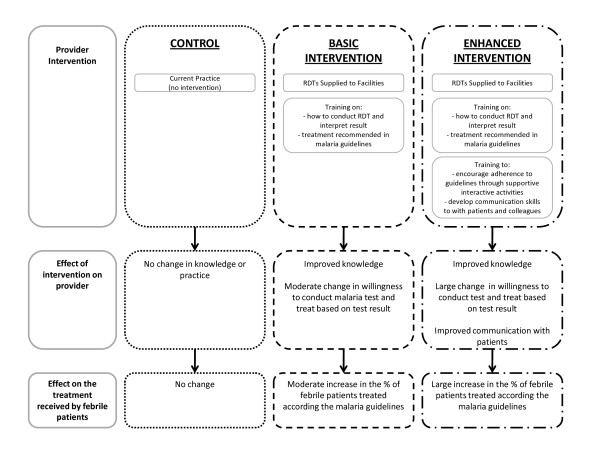
**Effect of Provider Interventions on the Treatment Received by Patients**.

The relationship between the study hypotheses and outcomes are summarised in Figure 1.

### Outcomes

#### Primary outcome

The primary outcome is the proportion of patients attending facilities that report a fever or suspected malaria and receive treatment according to malaria guidelines. The corresponding measure of cost-effectiveness is the cost per febrile patient that receives treatment according to the malaria guidelines.

Treatment according to the malaria guidelines is a composite endpoint requiring that:

• Febrile patients should be tested for malaria, using either microscopy or an RDT

• The patient should receive an ACT if he/she has a positive malaria test result

• The patient should not receive an antimalarial if he/she has a negative malaria test result

**Figure 2:**
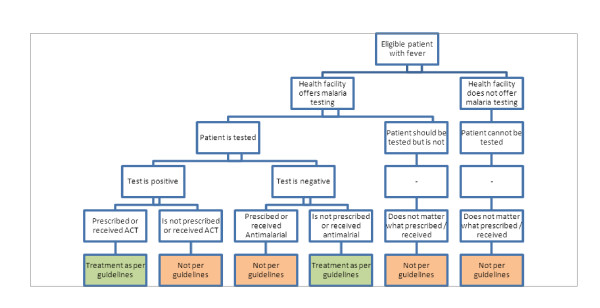
**Primary outcome measure**.

The outcome measure is summarized in Figure 2.

#### Secondary outcomes

Secondary outcomes include:

• Proportion of febrile patients that are tested for malaria

• Proportion of febrile patients receiving an antimalarial that receive an ACT

• Proportion of febrile patients receiving an ACT that receive the correct dose for their age

• Proportion of febrile patients receiving an ACT that accurately report how to take the medicine

• Proportion of febrile patients that report they are satisfied with the care received

• Proportion of HWs that report they were satisfied with the training received

• Proportion of HWs that know ACT should be given if the malaria test is positive and that an antimalarial should not be given if the malaria test is negative

• Proportion of HWs that report febrile patients should be tested for malaria

• Proportion of HWs that know how to identify positive, negative and invalid malaria RDT results

• Proportion of HWs that know the correct dose of the first-line ACT in an adult and in a child aged 2 years

• Total cost of the provider interventions and the cost per HW attending the BT and ET

Secondary outcomes related to patients will also be reported in terms of their urban/rural residence and socioeconomic status.

### Evaluation design

The evaluation of the intervention will use data collected in a patient exit survey, a register of malaria tests conducted by the provider during patient consultations, a provider survey, documentation of the intervention process, costing of the intervention activities and lastly, independent testing of malaria by the study team (see 'quality assurance'). The patient exit survey will be administered before the provider survey to ensure that the treatment received by patients is not influenced by the content of the provider questionnaire. Each of these is described below.

#### Patient exit survey

The primary outcome will be measured through an interviewer-administered patient exit survey. Data collection will commence three months after the intervention has been implemented. The three-month lag in the data collection is to ensure that the effect measure reflects treatment practices in the medium-term. In the short-term it is recognised that it is possible that the effect is overstated because health workers may change practices initially but revert to past behaviours over time, or that the effect is understated because it takes time for the training to have an effect as some health workers are hesitant and want to learn from the experience of the early-adopters. The survey data collection will take up to two months and will be organized such that the data will show the effect of the intervention over this time period by establishing a maximum number of patients that can be surveyed each week.

The research team will recruit field workers and provide training over a week on all aspects of data collection related to the patient exit survey. The training will include a practical assessment of their ability to provide information to respondents about the survey, obtain consent and administer the questionnaire. The research team will supervise the field workers and will accompany the field worker at the start of data collection to obtain consent from the head of the facility and ensure the fieldworker adheres to the standard operating procedures. Supervisory visits to monitor the performance of the field workers will take place at least once each week during the data collection period.

The patient exit questionnaire is designed to collect information about the patient's experience of seeking treatment and has been piloted a selected facilities in the study site. The questionnaire contains the following ten modules:

A. Background Information, Consent and Screening Questions

B. Details of the Respondent and/or Patient

C. Reasons for attendance

D. Consultation and diagnosis

E. Treatment prescribed and received

F. Patient satisfaction and knowledge of malaria

G. Costs of seeking treatment

H. Household characteristics

I. Malaria test completed by the study team (in sub-sample of patients)

J. Malaria test completed by health workers (from register of malaria tests at facility)

#### Register of malaria tests conducted

The patient exit questionnaire will be supplemented by a register of malaria tests at each participating health facility because patients may not always know if they were tested for malaria and the result of the malaria test. With consent from the head of the facility, health workers responsible for conducting malaria tests will be asked to keep a register of all malaria tests undertaken. The following data will be collected: details of the patient, availability of microscopy and RDT, method of test conducted, test result and the provider that conducted the test. At each facility the field workers will collect the register of malaria tests at least once each week and will use the patient's name, gender, age, date of visit to identify the patients that completed the survey and record the details in Section J of the questionnaire.

#### Provider Survey

The research team will administer a provider survey to all providers responsible for the diagnosis and treatment of suspected cases of malaria. Providers are eligible to participate if their responsibilities include any of the following activities: taking patient signs and symptoms, undertaking diagnostic tests, prescribing or dispensing medication. Written informed consent will be obtained before commencing the survey.

The provider survey has been designed to collect data on the providers' characteristics, knowledge and preferences for diagnosing and treating malaria and details of the resources available at the health facility. The survey will be piloted with providers at facilities that are not participating in the study. The questionnaire contains the following modules (of which A-B are completed by all providers and C-G are completed once for each facility):

A. Background information, consent and screening questions

B. Health worker characteristics and treatment practices

C. Details of the health facility

D. Management and procurement of drugs

E. Availability of RDTs

F. Availability of Antimalarials

G. List of all health workers that are involved in diagnosis or treatment

#### Documentation of the Intervention Process

The implementation of the malaria training workshops delivered to health workers will be documented. Details of all participants attending the training course will be recorded. Participants will undertake a pre- and post-training test to determine the impact of the training on their knowledge of malaria diagnosis and treatment. All participants will be invited to complete the training evaluation, which assesses the content and delivery of the training course. In addition, the trainers will complete a form to record any challenges faced in running the training workshop. Finally, the process of distributing the RDTs to health facilities will be monitored and any problems with the procedures for replenishing RDT stocks will be documented.

#### Costing

Direct and indirect costs of each phase of the interventions (i.e. development, implementation, upkeep) will be assessed from both a provider and societal perspective using standard economic evaluation methodologies [32]. Cost data will primarily be estimated from health facility records, project financial accounts and from the provider and patient exit surveys. Any health care savings will also be included and subtracted from costs.

#### Quality assurance

##### Data collection and management

There is a quality assurance officer responsible for ensuring all implementation and evaluation activities adhere to standard operating procedures. Quality assurance will include monitoring the process of obtaining consent, data collection, transfer of completed survey instruments, data management and the secure storage of study materials. In addition, field supervisors will monitor the survey administration undertaken by field workers and make frequent visits (at least once a week) to assess the quality of data collection and review completed questionnaires.

Only authorised staff with appropriate training will have access to the databases to perform data entry. All databases will be password protected. Each data form will be entered by two data entry clerks in a database of the same structure using two different computers. Entries will be compared for discrepancies using the Epi info 2000 data compare utility. Any discrepancies will be corrected by crosschecking against the corresponding original questionnaire. Checks (validation rules) will be implemented in different fields of the database. Data will also be queried electronically to ensure the correct data is entered under the correct variables for each section of the form/questionnaire. A log of all data changes will be kept. Questionnaires will be kept in a locked filing cabinet.

##### Independent verification of malaria tests conducted and test results

Reliance on providers register of malaria tests conducted and their interpretation of the test result may be a risk for data quality. For example, we are dependent on the providers' skills in conducting and interpreting the test results and the accuracy of their record-keeping. We will examine the accuracy of the register of malaria tests by comparing the patient reported data on whether they had a test with the register. We will also independently conduct RDT tests in a sub-sample of 5% of patients on exit that reported they were tested for malaria to determine the degree of consistency between the test result recorded by the provider and the test result conducted by the fieldworker. In addition, a sample of cases (both positive and negative) will be tested using PCR to check the sensitivity and specificity of both RDT and Microscopy. Quality assurance of the RDTs is beyond the scope of the study.

### Sample size

#### Patient exit survey

Sample size calculations are based on the primary outcome, the proportion of patients that receive treatment according to malaria treatment guidelines. Based on results from the formative research we expect that this will be 15% in the control arm (current practice) with a coefficient of variation (*k*) within stratum of 0.3.

To evaluate the effect of each of the intervention arms compared to current practice we have powered the study to detect a 15% increase over the control, from 15% to 30%, which was deemed to be the minimum increase for each of the interventions to be worthwhile. Using methods for stratified cluster randomised trials [33] and assuming k = 0.25 in the intervention arms, 7 clusters per arm and 100 patients per facility are required to detect this improvement with 80% power at a 5% significance level. With allowance for drop outs from the trial we propose 9 facilities per arm. A lower coefficient of variation was assumed in the intervention arms due to the shared training.

If both intervention arms prove to be significantly better than the control it is likely that the enhanced intervention will be better than the basic intervention and we expect a further 10% improvement in the primary outcome, to 40%. Therefore to determine whether or not the basic intervention should be recommended we wish to evaluate whether it is just as effective as (i.e. non-inferior to) the enhanced intervention. Assuming that the largest difference between the two intervention arms that would be considered unimportant is 10% (i.e. non-inferiority margin) then using methods for equivalence in cluster randomised trials [33] 17 clusters per intervention arm with 100 patients per cluster are required to have 80% power to demonstrate that the limit of a one-sided 95% confidence interval (CI) will be 10% or less. With allowance for drop outs from the trial we propose 19 clusters per arm.

#### Provider survey

The sample size calculations for the provider survey gives the anticipated level of precision for calculating the proportion of providers that know the treatment guidelines (i.e. report that parasitological testing is recommended and that ACTs are for confirmed cases of malaria). Based on our formative research we can assume 3-4 providers per facility, an intra-correlation coefficient (ICC) of 0.1, and an estimate of the primary outcome in each arm of 90%. With 9 facilities in the control arm and 19 facilities in each of the intervention arms this allows us to estimate the true primary outcome with ± 11.8% precision in the control arm and ± 7.7% precision in each of the intervention arms.

### Randomisation

A total of 47 facilities, 23 in Bamenda and 24 in Yaoundé, will be randomised within stratum to receive current practice, the basic intervention (RDTs and basic training) or the enhanced intervention (RDTs with enhanced training). With cluster randomised trials there is an increased chance that the study arms are unbalanced with respect to known and unknown potential confounders, and therefore undermines the credibility of the trial results. Stratified randomisation will reduce the likely imbalance in factors known to be correlated with the study outcome and the study site. However, the current availability of microscopy and the type of facility (which will also capture variation in health worker and patient characteristics) were assumed to be important correlates and therefore a process of constrained, or restricted, randomisation [34,35] will also be implemented to balance these two factors across the study arms using data collected in the formative research.

Using restricted randomisation schemes increases the risk of producing a design which is biased and not valid. Moulton [35] describes a design as being biased if there is any difference across the clusters in their probability of allocation to any given treatment. A randomised design is said to be valid if every pair of clusters has the same probability of being allocated to the same treatment. If the design is not valid there is a risk that the Type I error changes from its nominal value of 0.05. We will assess the validity of the restricted randomisation by producing a matrix where the rows and columns represent the clusters and the elements of the matrix are the proportion of times each pair of clusters is allocated to the same study arm i.e. the probability that the i^th ^cluster is being allocated to the same intervention group as the j^th ^cluster. The matrix will then be examined for under- and over-represented pairs that would highlight any potential causes for concern in the randomisation.

Randomisation of the facilities will be performed by the study statistician after informed consent has been sought from the head of the facility to avoid selection bias. Patients (or caregivers) and fieldworkers administering the patient exit survey will be blinded to group assignment. The research team involved in implementing the training interventions and supervising data collection will need to be aware of which facilities receive the different interventions.

**Figure 3:**
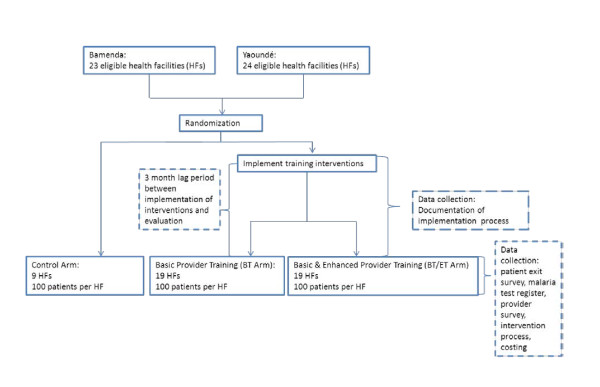
**Eligibility, selection, enrolment and methods of data collection**.

Figure 3 shows study eligibility, selection, enrolment and methods of data collection.

### Data analysis

Initially an overall test of the null hypothesis that there are no differences between either of the intervention arms and the control will be performed to guard against over-interpretation of any significant effects from individual comparisons of each intervention with the control, particularly if there is no evidence of a difference for any of the intervention arms.

The effect of the two interventions compared with control will be analysed with methods appropriate for cluster randomised trials. Point estimates of the primary outcome will be calculated using the unweighted mean of the cluster summaries in each stratum. If the distribution of the summary measures in each study arm is skewed, a logarithmic transformation to the proportions will be considered. An overall estimate of the risk ratio will be obtained by taking a weighted average of the stratum-specific risk ratios where the weights are inversely proportional to the stratum-specific variances. 95% confidence intervals (CI) will be adjusted for observed between-cluster variance and formal hypothesis testing will be conducted using stratified t-tests. Adjustment for covariates, including patient and provider characteristics and knowledge, contextual factors and process factors, will be carried out using a two-stage process. In the first stage, a logistic regression model including stratum as a fixed effect and the covariates of interest, but excluding the intervention effect, will be fitted to calculate cluster-specific expected values. The ratio of observed and expected values will be computed to give the ratio-residual for each cluster. In the second stage, the above methods for estimating the RR and 95% CI and hypothesis testing are carried out with the cluster-level proportions replaced with the covariate-adjusted residuals [35].

Non-inferiority between the two intervention arms will be assessed using the same methods as described above but instead of the risk ratio the risk difference will be estimated. Inference will be based solely on one-sided 95% CIs (or equivalently 2-sided 90% CIs).

Secondary outcomes on treatment received by patients, and provider knowledge and practice, will be analysed using the methods described above. To examine whether secondary outcomes vary according to the urban/rural residence and socioeconomic status of the patients methods appropriate for examining an interaction between the intervention and the individual-level variable will be applied [36]. Differences in coverage estimates between the intervention arms will also be estimated by calculating the arithmetic mean of the coverage proportions in each cluster and conducting a two-way analysis of variance, allowing for stratification.

For the economic analysis, cost-effectiveness ratios will be based on the primary outcome (i.e. the cost per case of suspected malaria that received treatment as recommended in the malaria guidelines) as well as a range of secondary outcomes including changes in provider knowledge. Cost-effectiveness will be calculated for each comparison and will be expressed as incremental cost-effectiveness ratios (ICERs). One-way and multi-way sensitivity analysis will be undertaken to examine the effects of varying uncertain variables on study findings. Costs and effects will be presented in both discounted and undiscounted form.

All data will be double entered using Microsoft Access 2007 (Microsoft Inc., Redmond, Washington) and analysed using STATA version 11.0 (STATA Corporation, College Station, Texas). A full analysis plan will be reviewed and agreed before the data are analysed.

### Trial status

Patients and providers are currently being recruited into the study for the patient exit survey.

## Discussion

Results from the study will be reported at local, national and international levels. At the local and national level, the Research on the Economics of ACTs (REACT) Project (http://www.actconsortium.org/pages/project-5.html) will continue working with the Ministry of Health after the trial is completed to adapt the most cost-effective interventions for national use. At the international level, we also see an opportunity to support the implementation of the 2010 WHO malaria treatment guidelines which acknowledge the need for provider training alongside the large-scale deployment of RDTs and ACTs.

## List of abbreviations

RDT: Rapid Diagnostic Test; ACT: Artemisinin-based Combination Therapy

## Competing interests

The authors declare that they have no competing interests.

## Authors' contributions

VW and WM secured the funding and are responsible for the overall study design and project management. OA and ANN were responsible for coordination and supervision of fieldwork. LM participated in the study design and overall study coordination. WM, OA and LM designed the provider interventions. BC led the statistical design. AMN coordinated data entry and management. CC contributed to study design and supervised the qualitative research component. All authors contributed to the original protocol while VW drafted the manuscript. All authors read and approved the final manuscript.

## References

[B1] Nshakira N Kristensen M Ssali F Whyte SR Appropriate treatment of malaria? Use of antimalarial drugs for children's fevers in district medical units, drug shops and homes in eastern Uganda Trop Med Int Health 2002 7 309 316 10.1046/j.1365-3156.2002.00858.x 11952946

[B2] Williams H Jones C A critical review of behavioral issues related to malaria control in sub-Saharan Africa: what contributions have social scientists made? Soc Sci & Med 2004 59 501 523 10.1016/j.socscimed.2003.11.010 15144761

[B3] Reyburn H Mbakilwa H Mwangi R Obeni M Raimos O Rapid diagnostic tests compared with malaria microscopy for guiding outpatient treatment of febrile illness in Tanzania: randomized trial BMJ 2007 334 403 10.1136/bmj.39073.496829.AE 17259188 PMC1804187

[B4] Zurovac D Njogu J Akhwale W Hamer DH Snow RW Translation of artemether-lumefantrine treatment policy into paediatric clinical practice: an early experience from Kenya Trop Med Int Health 2008 13 99 107 10.1111/j.1365-3156.2007.01980.x 18291008 PMC2592474

[B5] Zurovac D Tibenderana JK Nankabirwa J Ssekitooleko J Njogu JN Rwakimari JB Meek S Talisuna A Snow RW Malaria case-management under artemether-lumefantrine treatment policy in Uganda Malar J 2008 7 181 10.1186/1475-2875-7-181 18803833 PMC2556699

[B6] Rowe AK Ponce de Leon GF Mihigo J Santelli CFS Miller NP Van-Dunem P Quality of malaria case management at outpatient health facilities in Angola Malar J 2009 8 275 10.1186/1475-2875-8-275 19954537 PMC2795764

[B7] Juma E Zurovac D Changes in health workers' malaria diagnosis and treatment practices in Kenya Malar J 2011 10 1 10.1186/1475-2875-10-1 21214892 PMC3022768

[B8] Mangham L Cundill B Ezeoke O Nwala E Uzochukwu BSC Wiseman V Onwujekwe O Treatment of uncomplicated malaria at public and private health facilities in South-Eastern Nigeria Malar J 2011 10 155 10.1186/1475-2875-10-155 21651787 PMC3120734

[B9] Institut Nationale de la Statistique du Cameroun: Demographic Health Survey 2004 http://www.statistics-Cameroon.org

[B10] Cameroon National Malaria Control Programme. Annual Report 2008 http://www.minsante.gov.cm

[B11] World Health Organization Guidelines for the treatment of malaria 2010 second World Health Organization, Geneva

[B12] Mangham LJ Cundill B Achonduh OA Ambebila JN Lele A Metoh TN Ndive SN Ndong IC Nguela RL Nji AM Orang-Ojong B Pamen-Ngako J Wiseman V Mbacham WF Malaria Prevalence and Treatment of Febrile Patients Attending Health Facilities in Cameroon TMIH 2011, Nov 21 [Epub ahead of print] 10.1111/j.1365-3156.2011.02918.x 22098135

[B13] Ministry of Public Health of the Republic of Cameroon: Scaling up malaria control for impact in Cameroon. Global Fund Proposal (R9_CCM_CMR_HTM_PF_4Aug09_ENG), Geneva 2009 http://portfolio.theglobalfund.org/en/Grant/List/CMR

[B14] Hopkins H Asiimwe C Bell D Access to antimalarial therapy: accurate diagnosis is essential to achieving long term goals BMJ 2009 339 b2606 10.1136/bmj.b2606 19584113

[B15] Amexo M Tolhurst R Barnish G Bates I Malaria misdiagnosis: effects on the poor and vulnerable Lancet 2004 364 1896 1898 10.1016/S0140-6736(04)17446-1 15555670

[B16] Zikusooka CM McIntyre D Barnes KI Should countries implementing an artemisinin-based combination malaria treatment policy also introduce rapid diagnostic tests? Malar J 2008 7 176 10.1186/1475-2875-7-176 18793410 PMC2556342

[B17] World Health Organization Global Plan for Artemisinin Resistance Containment 2011 Geneva: Global Malaria Programme

[B18] Bell D Perkins MD Making malaria testing relevant: Beyond test purchase Trans R Soc Trop Med Hyg 2008 102 1064 6 10.1016/j.trstmh.2008.05.007 18586290

[B19] Williams HA Causer L Metta E Malila A O'Reilly T Abdulla S Kachur P Bloland PB Dispensary level pilot implementation of rapid diagnostic tests: an evaluation of RDT acceptance and usage by providers and patients - Tanzania, 2005 Malar J 2008 7 239 10.1186/1475-2875-7-239 19019233 PMC2613413

[B20] Chandler CIR Whitty CJM Ansah EK How can malaria rapid diagnostic tests achieve their potential? A qualitative study of a trial at health facilities in Ghana Malar J 2010 9 95 20398262 10.1186/1475-2875-9-95 PMC2859355

[B21] Kyabayinze DJ Asiimwe C Nakanjako D Nabakooza J Counihan H Tibenderana JK Use of RDTs to improve malaria diagnosis and fever case management at primary health facilities in Uganda Malar J 2010 9 200 10.1186/1475-2875-9-200 20624312 PMC2914063

[B22] Mukanga D Tibenderana JK Kiguli J Pariyo GW Waiswa P Bajunirwe F Mutamba B Counihan H Ojiambo G Kallander K Community acceptability of use of rapid diagnostic tests for malaria by community health workers in Uganda Malar J 2010 9 203 10.1186/1475-2875-9-203 20626863 PMC2914066

[B23] Onwujekwe O Uzochukwu B Dike N Uguru Nm Nwobi E Shu E Malaria treatment perceptions, practices and influences on provider behaviour: comparing hospitals and non-hospitals in south-east Nigeria Malar J 2009 8 246 10.1186/1475-2875-8-246 19863803 PMC2775747

[B24] World Health Organization Use of Rapid Diagnostic Tests 2006 Second ISBN 9290612045

[B25] Chandler CIR Mangham L Njei AN Achonduh O Mbacham WF Wiseman V As a clinician, you are not managing lab results, you are managing the patient: how the enactment of malaria at health facilities in Cameroon compares with new WHO guidelines for the use of malaria tests SS&M 2012 in press Unpublished data 22430000 10.1016/j.socscimed.2012.01.025

[B26] Smith LA Jones C Meek S Wenster J Review: Provider practice and user behaviour interventions to improve prompt and effective treatment of malaria: do we know what works? Am J Trop Med Hy 2009 80 3 326 335 19270276

[B27] Goodman CA Brieger W Unwin A Mills A Meek S Greer G Medicine Sellers and Malaria Treatment in Sub-Saharan Africa: What Do They Do and How Can Their Practice Be Improved? Am J Trop Med Hy 2007 77 Suppl 6 203 218 PMC2657822 18165494

[B28] Brieger W Unwin A Meek S Greer G Interventions to improve the role of medicine sellers in malaria case management for children in Africa 2005 London UK and Arlington, Va, USA: The Malaria Consortium and BASICS for the United States Agency for International Development; prepared for the Roll Back Malaria's sub-group for Communication and Training and Malaria Case Management Working Group http://www.rollbackmalaria.org/partnership/wg/wg_management/docs/medsellersRBMmtgsubcommitteereport.pdf

[B29] Atangana J Bigoga JD Patchoke S Ndjemai HMN Tabue NR Nem TE Fonjo E Anopheline fauna and malaria transmission in four ecologically distinct zones in Cameroon Acta Trop 2010 115 1-2 131 136 10.1016/j.actatropica.2010.02.014 20206111

[B30] Cameroon National Malaria Control Program Plan Strategique National de lute contre le Paludisme au Cameroun 2011 http://www.afro.who.int/en/downloads/cat_view/1501-english/969-countries/979-cameroon.html

[B31] World Health Organisation, FIND, CDC, & TDR Malaria rapid diagnostic test performance. Results of WHO product testing of malaria RDTs: Round 2 (2009) 2010 http://www.wpro.who.int/sites/rdt/who_rdt_evaluation/call_for_testing_round2.htm

[B32] Drummond MF Sculpher MJ Torrance GW O'Brien BJ Stoddart GL Methods for the economic evaluation of health care programmes 2005 3 Oxford: Oxford Medical Publications

[B33] Hayes RJ Moulton LH Cluster Randomised Trials 2009 Chapman & Hall/CRC

[B34] Sismanidis C Moulton LH Ayles H Fielding K Schaap A Beyers N Bond G Godfrey-Faussett P Hayes R Restricted randomisation of ZAMSTAR: a 2 × 2 factorial cluster randomized trial Clin Trials 2008 5 316 3278 10.1177/1740774508094747 18697846

[B35] Moulton LH Covariate-based constrained randomization of group-randomized trials Clin Trials 2004 1 297 305 10.1191/1740774504cn024oa 16279255

[B36] Cheung YB Jeffries D Thomson A Milligan P A simple approach to test for the interaction between intervention and an individual-level variable in community randomized trials TMIH 2008 19 2 247 255 10.1111/j.1365-3156.2007.01997.x 18304272

